# Prune Belly Syndrome with Overlapping Presentation of Partial Urorectal Septum Malformation Sequence in a Female Newborn with Absent Perineal Openings

**DOI:** 10.1155/2014/746323

**Published:** 2014-12-09

**Authors:** Azhar Farooqui, Alaa AlAqeel, Zakaria Habib

**Affiliations:** ^1^College of Medicine, Alfaisal University, Riyadh 11533, Saudi Arabia; ^2^College of Medicine, King Saud University, P.O. Box 2454, Riyadh 11451, Saudi Arabia; ^3^Department of Paediatric Surgery, King Faisal Specialist Hospital and Research Center, Riyadh 12713, Saudi Arabia

## Abstract

Prune belly syndrome (PBS) is a rare congenital anomaly characterized in males by a triad of anomalous genitourinary tract, deficient development of abdominal wall muscles, and bilateral cryptorchidism. Although similar anomalies have been reported in females, by definition they do not full fill the classical triad. Urorectal septum malformation sequence (URSM) is a lethal condition characterized by presence of ambiguous genitalia, absent perineal openings (urogenital and anal), and lumbosacral abnormalities. In this original case report, the authors discuss the presentation and management of what would be analogous to a Woodhouse category 1 PBS in a female newborn associated with an overlapping presentation of URSM.

## 1. Introduction

Prune belly syndrome (PBS), also called Eagle-Barrett syndrome or abdominal muscular deficiency syndrome, is a rare congenital disorder affecting 1 : 26,000 to 1 : 40,000 births, with a 97% occurrence in the male gender [1, 2]. The disorder in males is characterised by a triad of deficient abdominal muscle development, bilateral cryptorchidism, and a malformed urinary tract. Similar congenital malformations can be appreciated in females; however, with the absence of bilateral cryptorchidism, by definition they do not full fill the classic triad [3], and therefore they are often referred to as “pseudoprunes.” On the other hand, urorectal septum malformation sequence (URSM) is associated with ambiguous genitalia, absent perineal openings (urogenital and anal), and lumbosacral abnormalities. Herein, we report an extremely rare presentation of a female infant presenting as a case of prune belly syndrome with an unusual association with URSM.

## 2. Case Report

**Figure 1:**
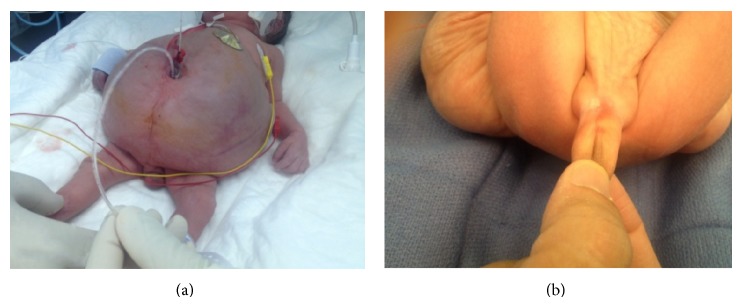
The abdomen was severely distended with signs of ascites (a). Also noted was ambiguous genitalia with a phallus like structure (b).

A 26-year-old primigravida presented to our emergency department with premature rupture of membrane with severe abdominal pain. An emergency cesarean section (C-section) was performed as the mother developed signs of preeclampsia, delivering preterm (34 weeks) female twins. Twin A was healthy and medically free with an unremarkable postnatal period. Twin B (birth weight 2.26 kg) needed to be intubated immediately as she failed to breathe spontaneously. APGAR score was 3 and 6 at 5 and 10 minutes, respectively. Initial examination revealed a markedly distended abdomen with signs of severe ascites (Figure 1). Perineal examination revealed a phallus like structure with no urethral, vaginal, or anal openings (Figure 1). Skeletal survey revealed a club foot deformity. The patient was shifted to the neonatal intensive care unit (NICU) for close observation and further work-up.

In the NICU, the patient was started on high frequency oscillation. Routine laboratory investigations and radiological examinations were ordered. Chest and abdominal X-ray revealed an abnormal bell shaped thoracic cage, a large distended abdomen, with stomach and bowel loops displaced to the right side of the spine, and decreased aeration in both lungs. Immediate surgical management in the NICU directed by progressive respiratory insufficiency involved placement of a Foley catheter through the umbilicus, via the patent urachus up to the bladder. An abdominal drain was inserted and was noticed to drain 500 cc of ascitic fluid, later analysed to be as urine. Karyotyping revealed normal 46XX chromosomes.

**Figure 2:**
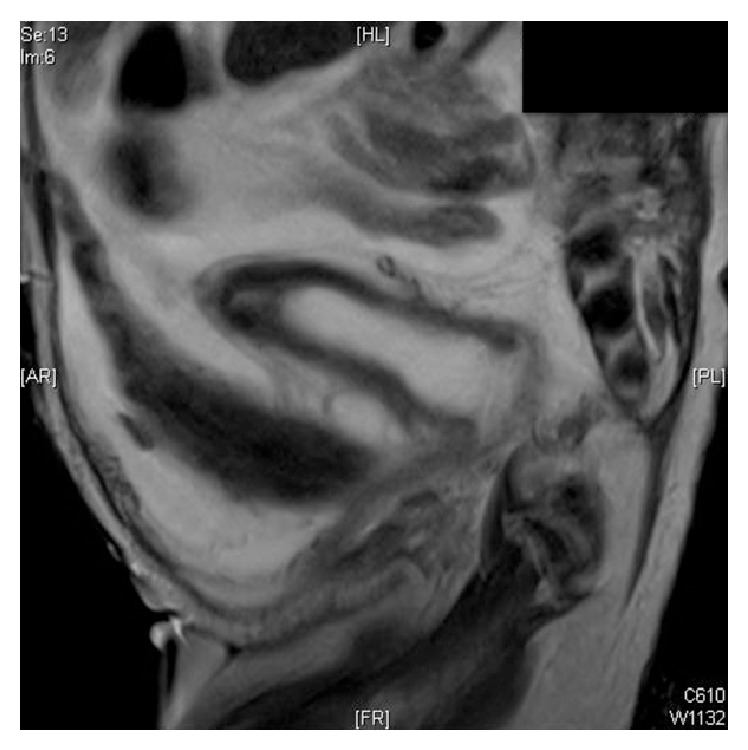
Pre-op MRI demonstrating an elongated bladder up to the level of urachus; a blind ending rectal pouch having no communication with the urogenital system; hydrometrocolpos; and absence of abdominal wall muscles.

Further radiological investigations (Figure 2) revealed hydrometrocolpos, absent anterior abdominal wall muscles, moderate hydronephrosis and hydroureter, and linear streaked calcifications in the left kidney which was smaller compared to the right (2.3 cm and 3.2 cm, resp.). A decision to do laparoscopic exploration was made.

Within the operating room, a temporary central line was established. Under general anaesthesia the patient was prepped and draped in supine position. A 5 mm trocar was inserted in the left upper quadrant, followed by 3 mm working ports. Exploration revealed an elongated midline bladder up to the level of the urachus, communicating with the umbilicus. There was a distended uterus with the fallopian tubes and ovaries present bilaterally. The colon was filled with meconium distally bulging into the pelvic cavity behind the uterus. Loop colostomy was secured; and the patient was transferred to the NICU in a stable condition. The hydrometrocolpos was managed by percutaneous drainage under interventional radiology. One month later, a vesicostomy was created to aid bladder drainage.

Patient passed away at the age of 3 months in the neonatal intensive care unit secondary to disseminated fungal urinary tract infection.

## 3. Discussion

Prune belly syndrome presents as a triad of anomalous genitourinary tract, deficient development of abdominal wall muscles, and bilateral cryptorchidism. The typical wrinkled appearance of the abdominal skin due to a defect in the muscles of abdominal wall often serves as a first clue to diagnosis. Urorectal septum malformation sequence (URSM) is associated with absence of perineal openings, ambiguous genitalia, urogenital, colonic, and lumbosacral anomalies.

Prune belly syndrome is a rare congenital disorder affecting 1 : 26,000 to 1 : 40,000 births, with a significantly higher prevalence in the male gender [1, 2]. In a study conducted by Baird and MacDonald [4], only 5 cases of prune belly syndrome were reported in females newborns (versus 13 in males) in over half a million consecutive life births recorded in British Colombia from 1964 to 1978.

PBS has been associated with involving congenital malformations in a variety of different body systems with 75% of these cases associated with cardiopulmonary, gastrointestinal, and orthopedic abnormalities. Club foot is present in 45% of the cases, with pulmonary hypoplasia, potter facies, imperforate anus, and arthrogryposis present in 45%, 27%, 27%, and 18% of cases, respectively [5]. Urologic abnormalities in PBS such as urethral hypoplasia or atresia are present in around 18% of cases and are independent risk factors for increased mortality [6].

Urorectal septum malformation sequence (URSM) is lethal in its full form [7]. However, partial URSM, which has been characterised by a single perineal opening draining into a common cloacae, has been reported to be compatible with life [8]. The reported incidence of URSM is between 1 : 50,000 and 1 : 250,000 [9].

The occurrence of prune belly syndrome associated with URSM in our case makes it an extremely rare entity, considering the extremely uncommon prevalence of these two conditions globally [1, 2, 9]. We identified only one other case in the literature with similar findings [3]. Goswami et al. reported a case of prune belly syndrome with URSM in a still born infant weighing 2.8 kg. However, the anatomical description presented by Goswami deferred significantly from the anatomical presentation provided in our case report. In our case, there was no communication between the blind ending rectal pouch and the urogenital system. The bladder was extending till the level of the urachus and the vagina did not have a perineal opening. Perhaps the patency of the urachus in our case served as a significant factor to increase the life span of the newborn.

Several studies have been published outlining the possible etiological factors responsible for the formation of prune belly syndrome. Reinberg et al. [10] suggested early urethral obstruction as a pivotal factor to the development of PBS leading to a resultant bladder distention, ureteral dilation, and hydronephrosis.

Genetic mutations have also been proposed to produce this clinical picture. Deletion of hepatocyte nuclear factor-1-beta gene at 17q12 has been linked to PBS in a couple of published case reports [11, 12]. This mutation has been associated with renal cysts, isolated renal dysplasia, and other malformations [11]. However, in 2012, Granberg et al. [13] concluded that functionally significant mutations of this gene are uncommon in PBS and advised further genetic studies to identify the genetic basis of this disease.

Familial cases of PBS have also been reported in the literature. Ramasamy et al. [14] reviewed 11 cases of familial PBS and proposed a sex-influenced autosomal recessive mode of inheritance. They reported 5 cases which appeared as autosomal recessive, 5 which appeared as X-linked recessive, and one which appeared as autosomal dominant.

The etiology of URSM has been linked to defects in mesodermal proliferation in early embryogenesis [7]. Kubota et al. [15] investigated cell proliferation and apoptosis in murine embryos that develop anorectal malformations secondary to overdose administration of long acting vitamin A analogues. Results of the study demonstrated defective cell proliferation and cell apoptosis in the cloacal membrane and dorsocaudal region on day 11 with lack of apoptosis in the anal orifice on day 12 suggesting the severity of the presentation is related to the age of the fetus when the developmental defect takes place.

Several researches have demonstrated a possible role of genetic mutations in the development of urorectal malformation sequence. Pennimpede et al. [16] demonstrated that in vivo knockdown of Brachyury (a key regulator of mesoderm formation during early development) produces anatomical defects including skeletal defects and URSM.

Treatment of prune belly syndrome primary involves surgical procedures to correct the undescended testes, reconstructing the urinary tract and abdominoplasty [17, 18]. Due to a significant prevalence of end stage renal disease in the prune belly population [19], kidney transplantation [20] has also been proposed as part of the management.

## 4. Conclusion

In this paper the authors describe an extremely rare case of prune belly syndrome in a female newborn presenting with overlapping symptoms of urorectal septum malformation sequence. Surgical management as well as a brief literature review is presented.
